# Natural-Product-Mediated Autophagy in the Treatment of Various Liver Diseases

**DOI:** 10.3390/ijms232315109

**Published:** 2022-12-01

**Authors:** Guifang Fan, Fanghong Li, Ping Wang, Xuejing Jin, Runping Liu

**Affiliations:** 1 School of Chinese Materia Medica, Beijing University of Chinese Medicine, 11 Bei San Huan Dong Lu, Beijing 100029, China; 2 Center for Evidence-Based Chinese Medicine, Beijing University of Chinese Medicine, 11 Bei San Huan Dong Lu, Beijing 100029, China

**Keywords:** natural products, autophagy, liver diseases, pharmacodynamics, scoring system

## Abstract

Autophagy is essential for the maintenance of hepatic homeostasis, and autophagic malfunction has been linked to the pathogenesis of substantial liver diseases. As a popular source of drug discovery, natural products have been used for centuries to effectively prevent the progression of various liver diseases. Emerging evidence has suggested that autophagy regulation is a critical mechanism underlying the therapeutic effects of these natural products. In this review, relevant studies are retrieved from scientific databases published between 2011 and 2022, and a novel scoring system was established to critically evaluate the completeness and scientific significance of the reviewed literature. We observed that numerous natural products were suggested to regulate autophagic flux. Depending on the therapeutic or pathogenic role autophagy plays in different liver diseases, autophagy-regulative natural products exhibit different therapeutic effects. According to our novel scoring system, in a considerable amount of the involved studies, convincing and reasonable evidence to elucidate the regulatory effects and underlying mechanisms of natural-product-mediated autophagy regulation was missing and needed further illustration. We highlight that autophagy-regulative natural products are valuable drug candidates with promising prospects for the treatment of liver diseases and deserve more attention in the future.

## 1. Introduction

Over the past several decades, liver diseases have become a global public health problem owing to their high prevalence and mortality rate. Particularly in China, which is known as the “leader in liver diseases”, over one-fifth of the population suffers from different types of liver diseases, such as chronic hepatitis B/C virus infection, alcoholic fatty liver disease (ALD), non-alcoholic steatohepatitis (NASH), non-alcoholic fatty liver disease (NAFLD), autoimmune liver disease, liver cirrhosis, hepatocellular carcinoma (HCC), and drug-induced liver injury (DILI) [1,2]. The spectrum of liver diseases has changed strikingly from viral hepatitis to predominantly comprising chronic metabolic liver diseases as the result of the development of novel diagnostic and therapeutic strategies as well as changes in living conditions. As one of the most common chronic liver diseases, fatty liver disease (FLD), including ALD and NAFLD, threatens the quality of life in about 25% of the world’s total population and causes a great economic burden to society [3]. It is characterized by the accumulation of a plethora of lipid droplets in hepatocytes and can progress from simple steatosis to steatohepatitis and subsequently to liver fibrosis, liver cirrhosis, and, eventually, devastating malignancies [4,5]. As a consequence of liver inflammation and regeneration, liver fibrosis (LF) is a reversible transition period from the original liver damage to liver cirrhosis. Normal liver tissues are gradually substituted by scar tissues and regenerative nodules in LF, which can progress to HCC [6]. As the final stage of diverse liver-related diseases, HCC is predicted to be sixth among the most commonly diagnosed cancers and the fourth leading cause of cancer-related death worldwide in 2018, with about 841,000 new cases and 782,000 deaths annually, according to GLOBOCAN 2018 produced by the International Agency for Research on Cancer [7]. According to the data from the Global Burden of Disease (GBD) study, China is regarded as one of the most high-risk areas in which the rate of age-standardized mortality and incidence of liver cancer is 2.1 times higher than the global average levels [8]. Moreover, multiple other liver disorder conditions are also widespread and cause extreme damage to human health.

Great progress has been made in understanding the pathophysiology and therapeutic strategies for diverse liver diseases. For example, targeting ER stress, oxidative stress, lipid metabolism, ferroptosis and fibrosis is conducive to the treatment of FLD. Unfortunately, the complexity of FLD makes the establishment of an effective modality for its management and the development of worthy therapies difficult. It seems that only weight loss and dietary control improve its histological characteristics, and FDA-approved drugs are still not available [9,10,11]. To slow the progress of HCC, many options are supplied by clinics, such as resection, transplantation, chemotherapy, targeted therapy, and radiotherapy, which are accompanied by significant shortcomings, including poor response, limited efficacy and unsatisfactory long-term prognosis [12]. Early diagnosis and interventional therapy, rational drug combination and controlled dosage are still common therapeutic strategies for LF and DILI, respectively. However, there remains a paucity of wonder drugs for curing liver diseases. Therefore, it is imperative to further decipher underlying pathogenic molecular mechanisms and develop putative therapeutic targets, as well as to discover potential drugs for different liver diseases.

Autophagy has gradually become a hotspot and has been implicated in a variety of diseases, including liver diseases, neurodegenerative diseases, chronic kidney diseases and bacterial or viral infections. As one of the major evolutionarily conserved housekeeping metabolic pathways originally discovered in the late 1950s [13], autophagy is a self-digesting process where cytoplasmic materials and organelles are sequestered into double-membrane autophagosomes and ultimately degrade in a lysosome-dependent manner, which then facilitates the recycling of damaged cellular components and maintaining the energy homeostasis [14]. Along with the ubiquitin–proteasome system, it is very important for autophagy to maintain a steady-state cellular metabolic process by compensating for intracellular and environmental stress conditions, such as nutrient starvation, protein aggregation, oxidative stress, lipid accumulation, hypoxia and the invasion of pathogens [15]. As an important metabolic organ in the body, the liver is abundant in lysosomes and possesses high levels of metabolic-stress-induced autophagy. The contributions of autophagy in providing starved cells with energy substances and promoting the clearance and renewal of damaged organelles are vital for the maintenance of hepatic homeostasis [16,17]. Many previous studies have proven that autophagy plays crucial and complicated roles in different liver diseases and represents an attractive therapeutic target [18].

As invaluable resources for innovative drug discovery, natural products have been long used for medical purposes due to their unique advantages exemplified by exceptional chemical and structural diversity, low toxicity and few side effects compared with chemical drugs. Statistics have demonstrated that more than 60% of the drugs that exist in the market were obtained from natural products, which also serve as lead compounds to achieve further structural optimization and derivation [19]. Until now, emerging evidence has shown that hundreds of natural products could regulate the physiological and pathological process of liver diseases via modulating autophagy, although misleading conclusions exist due to limitations and variations in experimental design. Moreover, there remains a lack of a systematic summary to overview the relationships between natural products, autophagy and liver diseases, as well as to evaluate the completeness and scientific significance of the literature. Herein, we aim to offer a critical and comprehensive review of natural product-originated autophagy modulators in the treatment of liver diseases and summarize approaches implicated in the current studies to provide novel insights into the improvement of future studies.

## 2. Overview of Autophagy and Its Regulation

**Figure 1:**
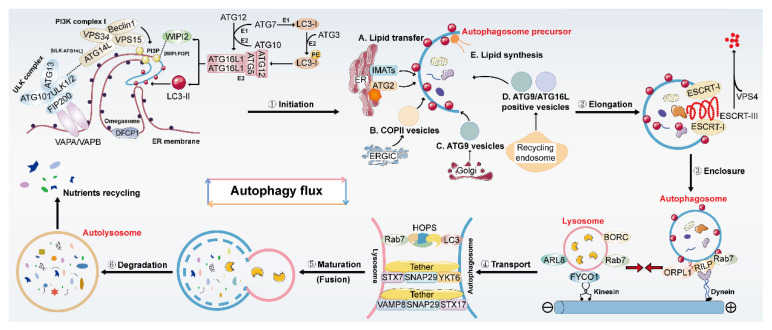
The detailed description of an intact autophagy flux in mammalian cells. A complete autophagy flux comprises multiple processes and occurs in a stepwise manner including: (1) the initiation of autophagosome precursor known as the phagophore; (2) the elongation of phagophore; (3) the enclosure of phagophore to form an enclosed autophagosome; (4) the transport of autophagosome and lysosome; (5) the autophagosome–lysosome fusion; (6) the degradation of autophagic substrates in autolysosome and nutrients recycling. Substantial protein complexes and signaling molecules are involved in and precisely regulated this process.

Autophagy is widely adopted by cells to combat diverse stresses and occurs in almost all eukaryotic cells. Owing to a different mode of the sequestration of degradation targets, autophagy can be divided into selective autophagy or non-selective autophagy. In the meantime, three subtypes of autophagy have been well-established, including macroautophagy, chaperone-mediated autophagy and microautophagy, which are named from their distinct mechanisms. Macroautophagy (referred to hereafter as autophagy) is the best-characterized autophagy form and has been studied well because of its prevalence of occurrence; therefore, next, we will simply summarize the current understanding of the mechanisms and molecular machinery of autophagy flux as well as its regulation in mammalian cells. Autophagy flux is referred to the continuous and complete progression of autophagy occurring in a stepwise manner (Figure 1) including multiple processes: (1) the initiation of autophagosome precursor known as the phagophore (also called isolation membrane); (2) the elongation of phagophore to surround the cargos for degradation; (3) the enclosure of phagophore to form the autophagosome (also called autophagic vacuole); (4) the anterograde and retrograde transports between autophagosome and lysosome; (5) the maturation of autophagosome characterized by fusion with lysosome or late endosome, and, later, the formation of autolysosome or endolysosome; (6) the subsequent degradation of autophagic substrates by lysosomal acid hydrolases for materials recycling [20].

Early studies demonstrate that in mammals, membrane compartments for autophagosome formation are primarily from the endoplasmic reticulum (ER), Golgi and the ER-mitochondria contact site. Among them, the specific ER domain containing DFCP1 (called the omegasome) is identified as a platform for the formation of phagophores on which PtdIns3P is abundantly produced from PI3K complex I. PtdIns3P is then employed to recruit the downstream molecules to trigger signaling cascades [21,22,23,24]. Furthermore, several autophagy-related (ATG) regulatory molecular components and complexes have been reported to be closely related to the modulation of each autophagy process. There are mainly two complexes encompassing the ULK complex and the PI3K complex I, and two ubiquitin-like conjugation systems including the ATG12-ATG5-ATG16L1 complex (refer to it hereafter as the ATG16L1 complex) and LC3-phosphatidylethanolamine (PE) conjugates are recognized to contribute to the precursor formation and nucleation. During the activation of autophagy, the ULK complex comprised ULK1/ULK2, ATG13, ATG101 and FIP200 is recruited to ER mediated by the interaction between VAPA/VAPB and FIP200, then served as a scaffold to recruit the downstream factors and regulates their functions [25]. In addition, the PI3K complex I which contains VPS34, VPS15, BECN1, and ATG14L can produce PtdIns3P in the omegasome which is correlated with autophagosome biogenesis by recruiting different PtdIns3P-binding proteins [26]. As one of the PtdIns3P-binding proteins, the ATG16L1 complex bound phosphoinositides 2b (WIPI2b) is recruited into the omegasome depending on the PtdIns3P binding of WIPI2b [27]. WIPI2b plays a vital role in lipid transport and functions as a ubiquitin E3 ligases, the ATG16L1 complex promotes the attachment of LC3 to PE on the membrane of phagophore, leading to LC3 lipidation coupled with the assistance of the E1-like ATG7 and the E2-like ATG3 enzymes [28]. It is well accepted that upon autophagy induction, cytoplasmic form LC3-I moves to the phagophore’s membrane and undergoes lipidation which finally converses to membrane type LC3-II. Thus, lipidation of LC3 is identified as a common hallmark of autophagy induction and is also essential in membrane expansion, transport and autophagosome–lysosome fusion through different mechanisms [29]. Additionally, the turnover of LC3 for the next round of autophagosome formation is accurately mediated by ATG4 which catalyzes the de-lipidation of LC3 [30]. In addition, as a ubiquitin-binding autophagy adaptor, P62 is supported to selectively bind to ubiquitinated autophagic degradation substrates and then be degraded in autolysosomes. Hence, the protein and mRNA abundance of LC3-II and P62 are generally considered as the basic indicators for determining whether autophagy is activated or not.

The next step of autophagy flux involves the elongation of the phagophore membrane which primarily relies on the delivery of lipids mediated mainly by three distinct mechanisms: membranes coming from the pre-existing organelles, vesicle-mediated lipid delivery and protein-mediated lipid transport. The ER is the main organelle for membrane expansion, and lipids located at the omegasome of ER are delivered to the phagophore through IMATs [31]. For vesicle-mediated delivery, COPII vesicles derived from the ERGIC are essential membrane precursors to transport lipids from ER to Golgi apparatus mediated by the ERGIC-ERES (the ER-exit site) contact, thus facilitating the membrane expansion [32,33]. In addition, ATG9 vesicles derived from the trans-Golgi network, ATG9- and ATG16L1-positive vesicles formed from recycling endosomes are also been implicated in phagophore elongation [34,35]. For protein-mediated lipid transport, WD-repeat proteins interacting with WIPIs, especially WIPI4, can connect with ATG2 at ER-phagophore junction and GRAM domain-containing 1A (GRAMD1A) to organize the phagophore elongation and size of autophagosome via transferring lipid membranes [36,37]. Moreover, mitochondria and de novo lipid synthesis also participate in the phagophore elongation [38,39]. During elongation, the precursor bends into a spherical shape, and the complete formation of the autophagosome by finishing the closure of the precursor is needed next. Based on previous studies, the ESCRT complex appears to be implicated in the accomplishment of this process. Assembly of complex and recycling of its components such as ESCRT-I subunit VPS37A, and ESCRT-III subunit CHMP2A are all responsible for fully achieving its function [40,41,42].

Upon the formation of a closed autophagosome, it moves and fuses with the lysosome. As lysosome is mainly located at perinuclear regions while autophagosome spreads throughout the cytoplasm, the anterograde transport of them mediated by kinesin motors and retrograde transport driven by microtubule motor protein dynein are indispensable for the following autophagosome–lysosome fusion. Previous studies suggested that multi-subunit BORC complex, GTPase Ras-related protein RAB7, and its effector FYCO1 play pivotal roles in the anterograde transport of lysosome by enabling them to meet and fuse with autophagosome [43,44]. RAB7 and its effectors ORP1L12 or RILP are proposed to enhance the retrograde transport of autophagosome and recruit the dynein-dynactin motor complex to lysosome [45]. Then, the fusion of autophagosome with lysosome involved the coordination action of the SNARE complex, RABs and tether proteins. In detail, two SNARE complexes including the STX17-SNAP29-VAMP8 complex and the YKT6-SNAP29-STX7 complex function redundantly to drive the process of fusion by directly binding autophagosomes and lysosomes with the help of other tethers such as EPG5, PLEKHM1, RAB7 and the HOPS complex. At the same time, the above tethers can promote autophagosome-lysosome fusion alone without the SNARE complex [14,46,47,48].

The final step of autophagy flux is the degradation of cargos inside of autolysosomal lumen, which depends on the activity of lysosomal acid hydrolases. Therefore, any factors that destroy the function of lysosome or break down its synthesis will markedly intercept the clearance of autophagic-lysosomal substrates and subsequently cause the over-accumulation of substrates which exhibits detrimental effects on the maintenance of balanced liver function. Since autophagy is considered as a promising therapeutic target for several human diseases, a plethora of autophagy activation and inhibition strategies are being explored and many drugs have been approved and applied in the study, such as the well-known mTOR inhibitor rapamycin (RAPA), potent AMP-activated protein kinase (AMPK) activators metformin, selective PI3K inhibitor 3-methyladenine (3-MA), lysosomal inhibitors chloroquine (CQ) or bafilomycin A1 (BafA1) [49]. Nevertheless, the involvement of autophagy in hepatic pathophysiology the and development of more autophagy regulators for the treatment of liver diseases are still displaying marked potential for further study.

Multiple stages and long processes are involved in a complete autophagy flux; thus, it is substantially challenging to accurately monitor the autophagy flux and evaluate the regulatory effects of drugs on autophagy. Since the transmission electron microscope (TEM) was first employed to identify autophagy in the 1970s, hitherto it has always been an indispensable method to visualize the ultrastructural morphology of autophagy structures and has long been termed as a golden standard to evaluate the autophagy occurrence [50]. Afterwards, more and more methods are developed for analyzing autophagy along with more multiple autophagy-related genes and proteins are discovered. Basic biochemical analysis methods such as RT-PCR, immunohistochemistry (IHC), immunoblot, and immunostaining analysis are utilized to analyze the expression of the autophagy markers LC3, P62, Beclin1, and others. To dynamically measure the autophagy flux, biosensors based on the fluorescent protein and colocalization analysis of different markers are performed to monitor autophagy at the single-cell level [51]. Tandem mCherry-GFP-LC3 autophagy reporter and other reporters with similar design principles (termed as autophagy tandem probe latter) are the usual biosensors. Among them, GFP is more sensitive to lysosomal acid cavities than mCherry; thus, it is GFP rather than mCherry that can be rapidly quenched in autolysosomes. Therefore, autophagosomes are shown as yellow puncta (mCherry^+^ and GFP^+^), whereas red puncta usually represent autolysosomes (mCherry^+^ and GFP^-^). Then, autophagy flux can be estimated well by detecting the amount and ratios of yellow and red puncta. The marked enhancement and increase in red fluorescence present autophagy activation and finishing, whereas the accumulation of yellow fluorescent spots can result from either increased upstream autophagosomes formation or impaired downstream autophagosome–lysosome fusion and degradation of autophagosomes which needed to be further confirmed and excluded using other methods. Furthermore, strategies for genetic and pharmacological induction or inhibition of autophagy are collectively executed to further clarify the effects of autophagy on diverse liver diseases. With the advancement of theoretical researches and technologies, several cutting-edge methods have been proposed and applied in studies. No individual assay mentioned above is perfect; thus, it is necessary to combine kinds of assays to systemically evaluate the autophagy flux and regulatory effects of agents on autophagy.

In recent years, many of natural products were reported to exert autophagy regulation properties in which a small part employed plenty of methods to comprehensively elucidate the effects of natural products on autophagy; however, existing evidence in most studies was still not adequate to clearly illuminate its regulatory function on autophagy. Therefore, related studies published from 2011 to 2022 were collected, and a score was given to each paper according to the rigidity of methodology for autophagy detection. Scoring criteria will be described in detail later. Based on this score, only a part of the studies which possess relatively reliable evidence to determine the influences of natural products on liver diseases by modulating autophagy will be reviewed next and is expected to provide innovative ideas for future research.

## 3. Potential Therapeutic Effects of Natural Products on Liver Diseases by Modulating Autophagy

### 3.1. Multiple Roles of Natural-Product-Mediated Autophagy Regulation in HCC

**Figure 2:**
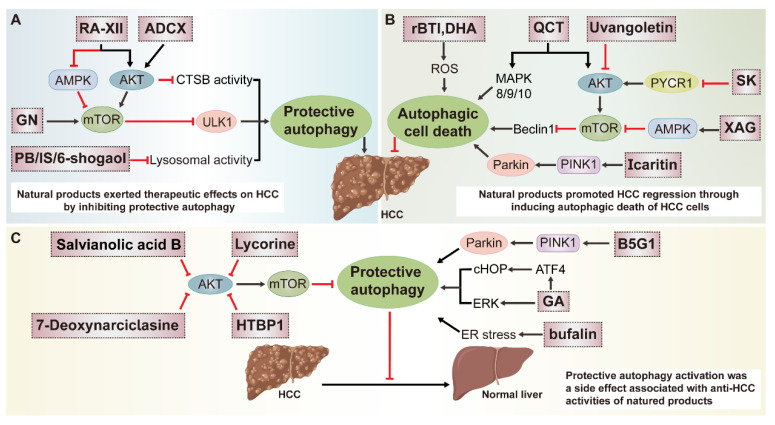
Multiple roles of natural-product-mediated autophagy regulation in HCC. (**A**) Natural products protect against HCC by inhibiting autophagy. ADCX, 25-O-acetyl-7,8-didehydrocimigenol 3-O-beta-D-xylopyranoside; PB, aqueous extract of *polygonum bistorta*; IS, icariside II. (**B**) Natural products exert anti-HCC effects by inducing autophagy. rBTI, Recombinant buckwheat trypsin inhibitor; QCT, quercetin; XAG, xanthoangelol; DHA, dihydroartemisinin; SK, Shikonin. (**C**) Autophagy activation is considered as a side effect associated with anti-HCC activities of natural products. GA, 18β-Glycyrrhetinic-acid.

Emerging evidence indicates that autophagy plays a bidirectional role during HCC development by either inducing hepatoma cells death or promoting their survival, which depends on the tumor microenvironment [16]. It is well accepted that recirculating energy substances from autophagy degradation can be utilized as major nutritional resources for the rapid proliferation of cancer cells. Autophagy activation antagonizes apoptosis and apoptosis-associated caspase activation blocks autophagy initiation in general. However, over-activated autophagy will degrade cytoplasm excessively which leads to autophagic cell death (ACD). As a novel type of programmed cell death, ACD is widely present in the liver and always companied by cells apoptosis. Many transcription factors such as p53 protein, BH3 (BCL-2 homology 3)-only proteins, Ser/Thr kinases and oncogenes are the common upstream signals of apoptosis and autophagy. They usually cross-regulate each other, yet responsible mechanisms of interplay between them have been partially but not fully elucidated in different diseases [52,53]. As described earlier, a panoply of natural products exert either pro- or anti-HCC properties in an autophagy-dependent manner, and regulations on autophagy, apoptosis or ACD exist intricately in this process. Due to the difference in hepatoma carcinoma cell lines or models and approaches employed to monitor the autophagy flux, varied conclusions are drawn in previous studies regarding the promotive or inhibitory effects of natural-product-regulated autophagy in HCC. In summary, the effects of natural products on autophagy in HCC are mainly divided into three categories described specifically next (Figure 2).

#### 3.1.1. Natural Products Protect against HCC by Inhibiting Autophagy

Recycling redundant proteins from autophagy supply energy for the malignant proliferation of cancer cells, hence disturbing autophagy is a potential therapeutic strategy against HCC. Recently, many natural products were identified to effectively restrain HCC growth by inhibiting autophagy at different stages (Figure 2A). Gomisin N (GN), a lignan isolated from dried fruits of *Schisandra chinensis* (Turca.) Baill, triggered apoptosis in HepG2 and HCCLM3 cells and inhibited autophagy initiation by disturbing the PI3K-AKT signaling, which subsequently decreased the activity of ULK1. A decrease in the protein levels of LC3-II and Beclin1 and an increase in P62 were observed after GN treatment. Enhanced LC3 accumulation induced by CQ was attenuated by GN which further determined the inhibitory effect of GN on the upstream of autophagy. Unfortunately, the definite relationship between autophagy and HCC cells apoptosis was not clearly elucidated [54]. As a unique natural cyclopeptide isolated from *Rubia yunnanensis*, RA-XII could inhibit protective autophagy for facilitating HepG2 cells apoptosis by modulating the AMPK/mTOR/P70S6K pathway. Both the protein and mRNA abundance of LC3-II and Atg3, 12, 16 were significantly reduced after RA-XII treatment; moreover, RA-XII-induced HCC cells apoptosis was greatly enhanced by CQ and compromised by RAPA [55]. Furthermore, Yu-Huei Liu et al. found that aqueous extract of *polygonum bistorta* L. (PB) induced apoptosis of Hep3B cells by compromising proteostasis. The up-regulation of LC3 and ATG12 and the inhibition of mTOR signaling after PB treatment were observed which meant the activation of autophagy. However, the up-regulation of P62 and down-regulation of Beclin1 (which was employed to indicate autolysosome in this study), as well as the accumulation of ubiquitinated EGFR caused by dislocation of the lysosomes were combined to indicate the obstruction of autolysosomes formation and lysosomal degradation induced by PB. The conclusion of this study is not rigorous enough since reversed authentication such as autophagy knockout or inhibition and other approaches including autophagy tandem probe, and TEM were absent to indicate the autophagy exactly. Furthermore, Beclin1 is frequently interpreted as one component of the PI3K complex which primarily contributed to autophagy initiation; therefore, it is unreasonable to select it as an autolysosome marker in this study [56].

Meanwhile, some other natural products also exerted regulatory effects on lysosomal function and subsequently implicated the degradation of autophagy substrates. For instance, 25-O-acetyl-7,8-didehydrocimigenol 3-O-beta-D-xylopyranoside (ADCX), a compound isolated from *Cimicifugae rhizoma* (Sheng-Ma in Chinese) could overcome multidrug resistance (MDR) during chemotherapy of HCC by inhibiting lysosomal degradation activity in liver cancer HepG2/ADM cells which in turn promoted ACD. As an eosinophilic fluorescent pigment, the fluorescence intensity of MDC can be measured to indicate the autophagic vacuole. They observed an increasing number of autophagic vacuoles after ADCX treatment as indicated by the GFP-LC3 assay and MDC staining. Additionally, the accumulation of protein LC3-II and P62 caused by ADCX were equivalent to cells challenged with BafA1, a selective (V)-ATPase inhibitor which inhibits the acidification of lysosomes and degradation of autophagy substances. We all know that deregulations of both the autophagosome–lysosome fusion and the degradative function of lysosome result in the deposition of autophagosomes or autophagy substances. In follow-up studies, the effect of ADCX on autophagosome–lysosome fusion was excluded as they observed that ADCX had no impact on the number of yellow puncta, and colocalization of LC3 (autophagosome marker) and LAMP1 (lysosome marker) under an autophagy tandem probe. As expected, although ADCX was unable to disturb the pH of the lysosome, whereas it destroyed the degradative activity of the lysosome which was characterized by lower red fluorescence intensity of DQ-BSA and the accumulation of autolysosomes with undigested substrates as detected by TEM. Moreover, when Atg5 was knocked out, ADCX-induced apoptosis was partly restored. Collectively, it is the impairment of the last stage of autophagy mediated by ADCX that induces ACD and improves multidrug resistance in HCC [57]. Similarly, a natural analogous to CQ, 6-shogaol isolated from ginger increased the sensitivity of Huh7 cells to tumor necrosis factor (TNF)-related apoptosis-inducing ligand (TRAIL) therapy by interrupting the last stage of autophagy flux. Exerting a similar function with CQ, 6-shogaol also culminated in the accumulation of protein LC3, P62 and autophagosomes. A combined treatment regimen with TRAIL and 6-shogaol or CQ could increase the protein levels of cleaved Caspase-3 and cleaved Caspase-8, suggesting that it was the inhibitory effects of 6-shogaol on autophagy that promoted Huh7 cells apoptosis [58]. Another study indicated that icariside II (IS) derived from *Epimedium koreanum* Nakai could induce apoptosis of HepG2 cells by impairing the function of lysosomes, but with no impact on the expression of autophagy initiation-related markers Beclin1, ATG3 and ATG5. The compromised lysosomal acidic environment and the loss of integrity of the lysosomal membrane were observed by AO staining which could indicate acidic vesicle organelles (AVO). The inhibition of lysosomal degradation induced by IS brought about enhanced accumulation of autophagy substrates LC3, P62 and damaged cellular organelles. Subsequently, IS-mediated autophagy inhibition and its therapeutic ability in HCC were further ascertained using the mouse-xenograft model in vivo [59].

#### 3.1.2. Natural Products Exert Anti-HCC Effects by Inducing Autophagy

A previous study demonstrated that quercetin (QCT) inhibited the proliferation of human HCC cells both in vitro and in vivo at least partially by stimulating autophagy (Figure 2B). The inhibition of the AKT/mTOR pathway and the activation of the MAPK pathways are considered to as the primary mechanisms. The increased autophagic flux, increased LC3-II and decreased P62 protein levels upon treatment with QCT were measured by biochemical, microscopy and structural methods in HCC cells. Additionally, co-treated with QCT and autophagy inhibitors hydroxychloroquine (HCQ) which blocks autophagosome–lysosome fusion could promote the accumulation of protein LC3-II and P62, yet another early inhibitor of autophagy 3-MA could reverse QCT-induced up-regulation in the ratio of LC3-II/LC3-I. Meanwhile, the dependence of ACD and growth inhibition of SMMC7721 tumors on QCT-induced autophagy was further confirmed by simultaneous treatment with HCQ which obviously attenuated the antitumor activity of QCT [60]. Similarly, a dihydrochalcone extracted from *Sarcandra glabra* (Thunb.) Nakai, uvangoletin exhibited antitumor effect in HepG2 xenograft model in vivo, and anti-EMT and pro-apoptosis effects in HepG2 cells in vitro by blocking the Akt/mTOR signaling to trigger autophagy. When autophagy was blocked, uvangoletin-induced apoptosis was significantly decreased, and the migration and invasion of cancer cells were remarkably recovered, which further confirmed the pivotal role of autophagy induction in the anti-tumor effects of uvangoletin [61]. Shikonin (SK), derived from Chinese herbal plant *Lithospermum erythrorhizon*, promoted HCC cells apoptosis and autophagy by down-regulating the expression of Pyrroline-5-carboxylate reductase 1 (PYCR1) which is involved in tumorigenesis. The effect of siPYCR1 on compromising the PI3K/Akt/mTOR signaling pathway and reinforcing autophagy initiation in HCC cells was observed, which further confirmed the regulatory role of PYCR1 in SK-mediated autophagy induction [62]. Moreover, xanthoangelol (XAG), isolated from *Angelica keiskei* (Miq.) Koidz. Exerted anti-HCC properties as indicated by suppression of HCC cells metastasis partially via activating autophagy in an AMPK/mTOR signaling-pathway-dependent manner. When treated with XAG, they observed stronger fluorescent signals in AO staining, more green and red puncta in autophagy tandem probe assay, increased protein levels of LC3II and Beclin1 and decreased P62 protein levels in Hep3B and Huh7 cells. CQ could further promote the up-regulation of LC3-II and P62, which testified to the promotive effects of XAG on autophagy occurrence. Moreover, the suppression of XAG on HCC cells migration, invasion, and epithelial-mesenchymal transition (EMT) mediated by activating the AMPK/autophagy pathway was largely reversed by 3-MA or knockdown of Beclin1 [63]. Additionally, almost identical methods were adopted to demonstrate that saikosaponin-d (SSd) could facilitate the inhibitory effect of radiotherapy on SMMC-7721 hepatoma cells proliferation by inducing autophagy. Autophagy inhibitor CQ or mTOR agonist can partially weaken the auxo-action of SSd to radiation-induced hepatoma cell death [64]. A previous study demonstrated that apoptosis and autophagy were simultaneously activated by dihydroartemisinin (DHA) from *Artemisia annua* L., which was mediated by a common upstream reactive oxygen species (ROS) production in HepG2215 cells. Suppression of caspase-1 by Z-YVAD-FMK decreased the expression of LC3-II and Beclin1, whereas inhibition of autophagy by CQ reduced the activation of caspase-1 in DHA-treated cells, manifesting that apoptosis and autophagy could regulate mutually. Additionally, compared to the control group, more autophagosomes and autolysosomes were detected in the DHA-treated group as illustrated by TEM. Furthermore, DHA promoted the lipidation of LC3 and the degradation of P62, and both could be increased by CQ, indicating that DHA elicited autophagy initiation indeed. In conclusion, it was DHA-induced HCC cells apoptosis and autophagy that contributed to HCC regression [65]. Solamargine, a natural product found in *Solanum nigrum* L. was also identified to exert an anti-HCC effect by effectively inducing autophagy both in vitro and in vivo. Autophagy inhibition using BafA1 or siLC3B protected HCC cells from SM-induced apoptosis [66].

Researches have shown that many risk factors for HCC such as viral infections or alcohol abuse were able to promote carcinogenesis via augmentation of oxidative stress, and HCC cells typically engaged the autophagy upstream of ROS signaling in order to meet their demands for malignant proliferation [67]. Therefore, mitophagy, which removes the damaged mitochondria selectively, has become a promising therapeutic target in HCC. Recombinant buckwheat trypsin inhibitor (rBTI), extracted from tartary buckwheat was reported to depolarize the mitochondria and increase the release of ROS, causing mitochondrial oxidative stress damages and meanwhile activating the mitophagy in HepG2 cells. Autophagy induction was supported by MDC staining, and they found an increase in the number of green fluorescent punctate structures in rBTI-treated cells compared to the control group. Increased fluorescence intensity of pEGFP-LC3 caused by rBTI could be further enhanced by BafA1, suggesting the formation of autophagosomes was due to autophagy induction rather than the blockade of autophagic degradation. Intensified colocalization of pDsRed2-Mito and pEGFP-LC3 detected by immunofluorescence assay, the up-regulation of LC3-II, Beclin1, Atg5, the down-regulation of P62, mitochondrial proteins Cox IV, Tom20 and the formation of mitochondria inside an autophagosome structure in rBTI-treated HepG2 cells were collected to clarify the induction of mitophagy. Unfortunately, although rBTI could depolarize mitochondria and increase the ROS production, the crosstalk among rBTI-induced damage to mitochondria, mitophagy and HCC treatment was still not understood. Thus, it will be more integrated to reveal the relationship between autophagy and HCC if the development of HCC is analyzed in a context of mitophagy inhibition in this study [68]. In another study, researchers found that the immunoregulatory drug icaritin could inhibit HCC cells proliferation via promoting immunogenic cell death (ICD) and triggering mitophagy by activating the PINK1-Parkin pathway, a classical mitophagy regulating signaling cascade. When treated with icaritin, the protein levels of Atg5, Atg7, LC3-II, PINK1, Parkin and colocalization fluorescence signal of LC3 and Mito-tracker were increased, whereas the expression of P62 was down-regulated dose dependently. Icaritin subsequently promoted the autophagy-dependent ATP secretion, a common marker used to determine ICD, and icaritin-induced decrease in intracellular ATP concentration was strikingly recovered upon BafA1 treatment. They further demonstrated that icaritin synergized with doxorubicin to induce ICD and played an essential role in HCC treatment by remodeling the immune-suppressive microenvironment, yet the correlation of icaritin-induced autophagy activation with ICD was poor elucidated in this study [69]. Another study in which ER stress-mediated autophagy activation induced by penta-1,2,3,4,6-O-galloyl-β-D-glucose (PGG), derived from *Rhus chinensis* Mill and *Paeonia suffruticosa* exerted bidirectional action during the development of HCC. It showed that PGG could induce the senescent phenotype of HCC cells and facilitate cells cycle arrest in an autophagy-dependent manner. Protein expression analysis revealed a progressive increase in the ratio of LC3-II/LC3-I and a decrease in P62 in PGG-treated HepG2 cells, and BafA1 supplementation promoted the accumulation of these proteins. Furthermore, the inhibition of autophagy by 3-MA or Atg5 knockout attenuated UPR activation and subsequently blunted cell senescence. On the other hand, the activation of autophagy antagonized HCC cells apoptosis which was unfavorable for the antitumor effects of PGG. Taken together, whether PGG-activated autophagy is beneficial or harmful in the treatment of HCC mainly relies on who is more dominant between the senescence and apoptosis of cells [70].

Moreover, the regulatory effects of some natural products on autophagy are still controversial, which primarily attribute to the limited technical approaches and varied models applied in these studies. Bufalin, extracted from bufonid’s skin exerted proapoptotic capability by inducing autophagy in multiple HCC cells. The increase in the ratio of LC3-II/LC3-I, Beclin1, Atg5, Atg7, and Atg12 protein levels, the degradation of P62 and the formation of autophagy vesicles implied that bufalin induced autophagy. Moreover, bufalin-induced ACD was strikingly suppressed when autophagy was inhibited by 3-MA or CQ [71,72,73]. However, a study also showed that endoplasmic reticulum stress-dependent autophagy activation triggered by bufalin comprised its anti-HCC ability, and damaging autophagy by 3-MA, BafA1 or Atg5 siRNA accelerated bufalin-induced Huh7 and HepG2 cells death [74]. Furthermore, Tai et al. [75] found that berberine (BBR), existing in many plant species such as *Berberis aristata* and *Berberis aquifolium*, could induce HCV-infected Huh-7 cells death and simultaneously inhibit autophagy, which was only characterized by reduced expression of both LC3-I and LC3-II. BafA1 pretreatment augmented BBR-induced cell death and there was no any other evidence to distinguish whether BBR-modulated autophagy inhibition contributed to the above process. Paradoxically, Hou et al. [76] thought that BBR could induce ACD in SMMC7721 and HepG2 cells which was proved by the formation of autophagy vesicles and the enhancement of BBR-induced cell death was observed in combination with 3-MA. As mentioned above, both bufalin and BBR exhibited varied influences on autophagy in HCC which reminds us that more integrated experiments are required to be designed and more sufficient evidences are needed to be supplied to support the outcomes of a natural products on autophagy in HCC.

#### 3.1.3. Autophagy Activation Is Considered as a Side Effect Associated with Anti-HCC Activities of Natural Products

It has been reported that substantial natural products simultaneously inhibit HCC and trigger autophagy which antagonizes the apoptosis or promotes the proliferation of hepatoma cells (Figure 2C), suggesting that the blockade of autophagy may synergize the anti-cancer effects of these natural products. Multiple reports have suggested that mitophagy is conducive to drug resistance in cancer cells. B5G1, a derivative of betulinic acid, induced mitochondrial dysfunction and ROS overproduction which resulted in mitochondrial apoptosis and PINK1-Parkin-mediated mitophagy in MDR cancer cells HepG2/ADM and MCF-7/ADR rather than in their parent cells HepG2 and MCF-7. They observed that B5G1 induced the ubiquitylation of mitochondrial fusion protein Mfn2 and promoted the mitochondrial anchoring of autophagy adaptor P62, which was necessary for the recognition of damaged mitochondria by autophagy-related proteins and autophagic degradation. In detail, the colocalization of Mito-tracker and P62 was enhanced by B5G1 and the colocalization of Mito-tracker and LC3 was abolished upon P62 siRNA pretreatment. The enhancement of colocalization between Mito-Tracker and LC3/LAMP1/Lyso-Tracker, the up-regulation of the LC3-II/LC3-I ratio and PINK1, and the degradation of mitochondrial proteins COX IV, TOM20, Mfn2 after B5G1 treatment were observed after B5G1 treatment. siRNA-mediated knockout of PINK1 or Parkin instead of Beclin1 could abrogate the above phenomenon. When PINK1 siRNA, mdivi-1 (a mitophagy inhibitor) or BafA1 was supplemented to dampen the autophagy, both apoptosis and sensibility of multidrug-resistant cancer cells to B5G1 were counterbalanced which further highlighted the holding back effect of B5G1-induced mitophagy activation on its anti-HCC activities. Therefore, blocking mitophagy will sensitize MDR cancer cells to B5G1 [77]. In another study, 18β-Glycyrrhetinic-acid (GA), a main compound of *Glycyrrhiza uralensis* Fisch, was identified to elicit the effects of both apoptosis induction and autophagy activation on HCC cells. Using autophagy tandem probe, GA-induced autophagy was illustrated by significant increases in both yellow and red puncta in mRFP-GFP-LC3 stably transfected HepG2 cells, in addition to an increase in the ratio of LC3-II/LC3-I and P62 which were further notably accumulated in the presence of CQ. In the meanwhile, inhibiting autophagy pharmacologically using CQ or genetically by si-ATG5/ATG7 augmented GA-induced HepG2 cells apoptosis, whereas autophagy activator RAPA reduced their apoptosis to some extent which verified from both positive and negative aspects that GA-induced complete autophagy flux played a cytoprotective role in neutralizing its pro-apoptotic properties [78]. A similar result was observed in another previous study, in which lycorine extracted from *Lycoris radiata* (Amaryllidaceae) Herb promoted HCC apoptosis and induced protective autophagy. Moreover, lycorine-induced HCC cells apoptosis was remarkably promoted when autophagy was blocked pharmacologically and genetically [79]. Recently, Hongwei Liu et al. reported that an alkaloid isolated from *Tylophora ovata*, HTBPI (abbreviation) could also trigger protective autophagy to oppose HCC cells apoptosis, and the pretreatment with 3-MA or BafA1 could decay the inhibitory effect of HTBPI on the expression of protein P62 while facilitating the HTBPI-induced HCC cells apoptosis. Increase in the ratio of LC3-II/LC3-I, Beclin1, decrease in P62, the formation of substantial red puncta, and occasional yellow puncta under the observation of autophagy tandem probe were collected to support the effects of HTBPI on autophagy activation. The antitumor effect of HTBPI in vivo has also been evaluated in a patient-derived xenograft (PDX) model. In consistent with the in vitro data, HCC cells apoptosis and degradation of autophagy adaptor P62 were markedly increased, whereas the tumor size was decreased in the HTBPI-treated group in comparison with the vehicle-treated control group [80]. Taking advantage of nearly the same concept and experimental design, the repressive effects of 7-deoxynarciclasine, isolated from *Lycoris radiata* (Amaryllidaceae), on HCC progression and the detrimental role of 7-deoxynarciclasine-induced pro-survival autophagy in the above process were identified [81]. Many other natural products such as glycyrrhetinic acid, salvianolic acid B, acanthopanax senticosus harms extract and arenobufagin could also synchronously induce HCC cells apoptosis and protective autophagy which antagonized their anti-HCC properties. However, the conclusions will be more convincing if the interplay between apoptosis and autophagy are comprehensively investigated in these above studies [82,83,84,85].

### 3.2. Potential Application of Natural Products in Fatty Liver Disease (FLD) Treatment by Modulating Autophagy

**Figure 3:**
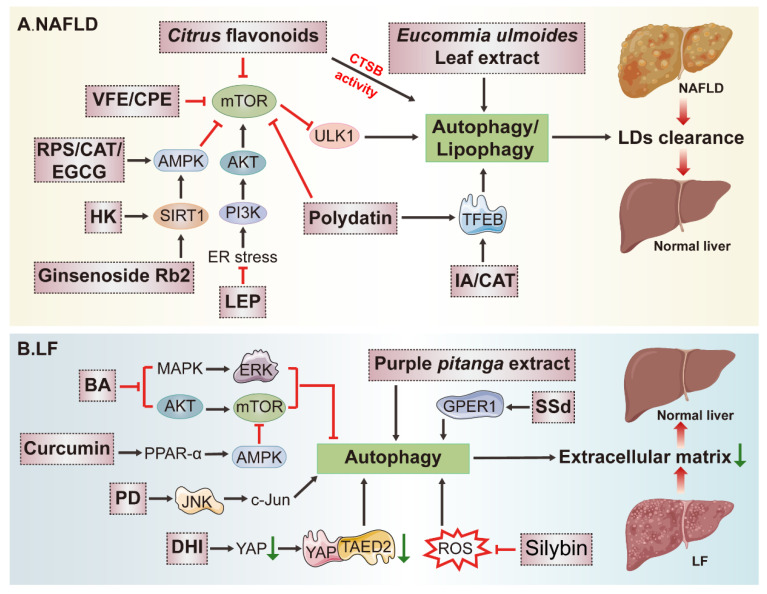
Potential application of natural products in the treatment of NAFLD and LF by modulating autophagy. (**A**) Natural products alleviated NAFLD by promoting clearance of over-accumulated LDs in an autophagy-dependent manner. VFE, ethanol extract of *Valeriana fauriei*; CPE, ethanol extract of *Cyclocarya paliurus*; RPS, the water extract of red peppers seed; CAT, catalpol; EGCG, Epigallocatech in-3-gallate; HK, honokiol; LEP, α-linolenic acid-enriched cold-pressed perilla oil; IA, isopropylidenyl anemosapogenin. (**B**) On the one hand, natural products decreased the deposition of excessive extracellular matrix by inducing autophagy. On the other hand, some comprised over-activated autophagy in LF to damage fibrosis progression. BA, betulinic acid; PD, Platycodin D; DHI, dihydrotanshinone I; SSd, saikosaponin-d.

As one of the most common liver diseases, FLD is broadly divided into NAFLD and ALD in terms of their pathogenesis; the latter is deemed to be less prevalent than the former, and autophagy in NAFLD will be emphatically reviewed next. By screening rare gene polymorphism in patients, Guido A Baselli et al. identified that ATG7 loss-of-function variants promoted NAFLD progression by damaging autophagy [86]. Both ATG5 deficiency in liver CD11c+ cells in mice and Rubicon-mediated autophagy inhibition were reported to accelerate NAFLD progress [87,88]. Emerging evidence has also demonstrated that autophagy is generally impaired in FLD, and consequently the activation of autophagy or lipophagy which degrades liver lipids (LDs) selectively is regarded as an effective strategy to clear the overwhelming deposition of LDs in the liver. Previous studies have suggested that many natural products could reduce aberrant or excessive lipid accumulation by triggering autophagy (Figure 3A).

The ethanol extract of *Valeriana fauriei* Briq (VFE) was proven to improve hepatic lipid accumulation in HFD (high-fat diet) mice and promoted clearance of LDs in oleic acid (OA)-treated Huh7 cells by inducing the mTOR/ULK1-mediated lipophagy, as evidenced by VFE-induced formation of autophagy-like structures and increase in the protein and mRNA levels of LC3-II and P62, which could be further accelerated by BafA1 or CQ, supporting for the autophagy activation. Moreover, as the same as Torin1 (the positive control which can induce autophagy by inhibiting the mTOR signaling pathway), VFE decreased LDs deposition and enhanced the colocalization of LC3-labeled autophagosomes and Bodipy-indicated LDs, which further verified the induction of lipophagy. Additionally, after being challenged with VFE, both HFD mice and lipotoxic hepatocytes recovered from impaired lysosomal activity along with the increase in cathepsins B (CTSB) activity. Collectively, VFE is greatly potential and promising to protect against NAFLD by affecting several stages of autophagy [89]. Similar technical approaches were adopted to ascertain that honokiol (HK) derived from the plants of *Magnolia* genus L. could alleviate lipid accumulation via Sirtuin3 (SIRT3)-AMPK-induced autophagy initiation in choline-deficient high-fat diet (CDHFD)-fed mice, and palmitic acid and oleic acid mixture (P/O)-treated AML12 cells. More red puncta were formed in the mRFP-GFP-LC3 assay, the colocalization of mRFP-LC3 and Nile red (indicated LDs) was increased, the P/O-induced shrinkage in the ratio of LC3-II/LC3-I and Beclin1 level, and the elevation of P62 were all reversed in HK-treated cells in comparison with the control. In addition, the LD fraction was isolated and the enrichment of LC3-II, Beclin1 and decreased P62 were observed in LD fractions rather than in whole cell homogenates after HK treatment, which further confirmed the lipophagy activation [90]. In another study, the ethanol extract of *Cyclocarya paliurus* (CPE) was reported to effectively induce lipid clearance associated with increased autophagy. Lipophagy activation was visualized by the increased colocalization of Lyso-Tracker red and Bodipy, the up-regulation of LC3-II and Beclin1, the degradation of P62, and the existence of autolysosomes structures in TEM images after CPE treatment in steatotic HepG2 cells [91]. In another study, a relatively pure LD fraction was isolated from the livers. To avoid the difference in amounts of LDs from different fractions, the LC3-II/ADRP ratio was employed to measure the LC3-II expression. It showed that LC3-II was mainly enriched in the LD fraction, whereas LD-free lysates largely contained LC3-I. The increase in the LC3-II/LC3-I ratio, Beclin1, as well as the decrease in P62 after supplementing with bergamot polyphenol fraction (BPF) from *Citrus bergamia* Risso et Poiteau were observed in response to excessive lipid accumulation in the liver of CAF diet-treated mice, indicating that lipophagy stimulation was the main target of BPF in alleviating NAFLD [92]. As previously suggested, α-linolenic acid-enriched cold-pressed perilla oil (LEP) could inhibit hepatic steatosis and recover the autophagy via PI3K/AKT/mTOR pathway in HFD mice liver. HFD-induced ER stress was reduced and the increased autophagy was reversed by LEP, but despite all that, the regulative effects of autophagy inhibition on hepatic steatosis were still undefined [93]. *Citrus* flavonoids, *Eucommia ulmoides* leaf extract and dioscin were also recognized to protect against NAFLD and meanwhile activate autophagy in various studies [94,95,96]. The recognition of autophagy is comprehensive in these above studies; nevertheless, it will be more satisfactory to evaluate the development of NAFLD when autophagy is suppressed, which reversely verifies the significance of autophagy induction in overcoming lipid damage in NAFLD.

Autophagy retardation caused by disorders of lysosomal degradation was observed in methionine-choline deficient (MCD) induced NASH model in db/db mice. Based on this model, the therapeutic potential of polydatin derived from *Polygonum cuspidatum* Sieb. et Zucc. in NASH by restoring lysosomal function and autophagic flux was investigated. They observed that PA or BafA1-induced damage of autophagosome–lysosome acidification was restored by polydatin or RAPA. Otherwise, lysosomal enzyme activities and the accumulation of LC3-II and P62 in the NASH livers were also rectified by polydatin. With respect to the mechanism, polydatin-induced TFEB activation and inhibition of mTOR signaling were primarily contributed to enhance autophagy initiation during the treatment of NASH [97]. Furthermore, the destructive autophagy flux in choline-deficient, L-amino acid-defined and high-fat diet (CDAHFD)-induced NASH mice was recovered by isopropylidenyl anemosapogenin (IA), which is isolated from the traditional Chinese medicine *Salvia miltiorrhiza* Bunge. The degradation of CDAHFD-induced accumulation of protein LC3 and P62, in addition to the activation of TFEB and increased lysosome activity, were observed in IA-treated group. Autophagy inhibition impaired IA-induced lipid clearance as indicated by Oil Red O (ORO) staining, suggesting the therapeutic effects of autophagy in NASH remission [98].

Recent advances have shown that AMPK, a crucial cellular energy sensor, is essential for the activation of the autophagy pathway in the pathogenesis of NAFLD by coordinately regulating ULK and VPS34 kinase complexes [99]. Epigallocatechin-3-gallate (EGCG), a polyphenol derived from green tea, has been recognized to contribute to LDs clearance in the liver by orchestrating the AMPK-dependent autophagy initiation. In HepG2 cells transfected with RFP-GFP-LC3 plasmid, they observed that EGCG increased both yellow and red dots in merged images, suggesting the occurrence of fusion and protein degradation within the lysosome. Increased autophagic flux in HepG2 Cells and mouse hepatocytes in primary culture was also identified, and intracellular lipid content determined by bodipy staining was notably decreased by EGCG which was almost abolished after ATG5 or AMPK knockdown, indicating the crucial role of autophagy in NAFLD repression [100]. Similarly, the water extract of red peppers seed (RPS) from *Capsicum annuum* L. alleviated hepatic lipid accumulation through AMPK-relied and autophagy-mediated down-regulation of lipogenesis in HFD-fed mice and OA-induced steatosis cells [101]. Moreover, the major bioactive ingredient of *Rehmannia* (Di Huang) *glutinosa* DC. catalpol (CAT) also exerted anti-hepatic steatosis properties and attenuated lipotoxicity-mediated apoptosis in NAFLD by inducing the AMPK/TFEB-modulated autophagy. CAT administration greatly decreased CQ-induced P62 accumulation, up-regulated the expression of autophagy-related genes and enhanced the formation of autophagic vacuoles in hepatocytes. The protective effects of CAT on lipid clearance and lipo-apoptosis were eliminated by co-treatment with CQ, which indirectly revealed the significance of CAT-induced autophagy in mitigating the development of NAFLD [102]. Similarly, a study deciphered that ginsenoside Rb2 (from *Panax ginseng* C.A.Mey.) eliminated lipid accumulation by rectifying defective autophagy in a Sirtuin1 (SIRT1)/AMPK pathway relied manner in db/db mice and cultured steatotic hepatocytes. The recovery of lipotoxicity-induced autophagic damages and lipid clearance after ginsenoside Rb2 treatment was abrogated in the presence of CQ [103].

Regarded as the ‘power house’ of cells, mitochondria are responsible for the aerobic respiration whose dysregulations are common performances in NAFLD. Therefore, exploiting mitophagy contributes to the maintenance of cellular homeostasis and holds promise as a therapeutic target in NAFLD. Data from patients and mice with NAFLD have shown that the protein abundance of two mitochondria proteins TOM20 and COX IV, autophagy markers P62 and LC3-II were higher whereas mitophagy-related Parkin and PINK1 were lower compared with healthy ones. It turned out that the impairment of PINK1-Parkin-dependent mitophagy could be effectively reversed by cyanidin-3-O-glucoside in NAFLD which was proved by autophagy tandem probe analysis, TEM and combined with detection to the levels of mitophagy-related proteins. Furthermore, the knockout of PINK1 markedly reversed the improvement of a spectrum of features about hepatic steatosis delivered by cyanidin-3-O-glucoside, demonstrating that mitophagy activation was crucial for cyanidin-mediated-lipid elimination in NAFLD [104]. In addition, corilagin existed in many *ethnopharmacological plants*, such as *Phyllanthus reticulatus*, *Geranium wilfordii*, *Phyllanthus emblica*, and *Dimocarpus longana*, could eliminate hepatic lipid over-storage, reverse defective autophagy and restore damaged mitochondria in HFD mice by triggering mitophagy [105]. Although majority of studies revealed that many natural products possessed anti-NAFLD activities by augmenting autophagy, some natural products were recognized to ameliorate NAFLD yet concurrently by retarding autophagy. For instance, lotus seedpod extract (LSE) improved lipid accumulation and simultaneously inhibited autophagy in OA-challenged HepG2 cells [106]. Although inhibitory effects of LSE on autophagy in NAFLD have been well elucidated, those of the underlying mechanisms such as how it relieves steatosis by inhibiting autophagy remains to be illuminated.

As a common risk factor of liver injury, ethanol was confirmed to trigger hepatocyte senescence, a key event participating in the progression of alcoholic liver disease, by decreasing the proteins expression of cellular communication network factor 1 (CCN1) and Sestrin2. Pterostilbene, a dimethylated analogue of resveratrol which widely exists in grapes, blackberries and blueberries could significantly reversed the above phenomenon in vivo and in vitro. Intriguingly, recovery of ALD-forced CCN1 up-regulation was essential for pterostilbene to execute its anti-senescent function as the over-expression of CCN1 restrained its anti-senescence effects on ALD. Mechanistically, pterostilbene-triggered CCN1 reduction was dependent on autophagy-mediated post-transcriptional regulation but not transcriptional regulation. Pterostilbene restored autophagic flux in damaged hepatocytes by increasing the expression of Sestrin2, a core upstream modulator of autophagy, which then promoted autophagy adaptor P62 to the anchor and resulted in the degradation of CCN1 in autolysosomes. The interaction between CCN1 and P62 was exhibited by co-immunoprecipitation (Co-IP) analysis, and co-localization of CCN1 with LAMP1 or LC3 was observed using immunofluorescence staining in this study, testifying the above mechanisms [107]. Limin Gao et al. also recognized that Kinsenoside (KD), an active ingredient extracted from *Anoectochilus roxburghii* (Wall.) Lindl. (Orchidaceae), alleviated ALD by reducing oxidative stress, while activating AMPK-dependent protective autophagy. They observed that KD led to the up-regulation of STRAD and LKB1, the upstream of AMPK, resulting in the up-regulation of proteins associated with autophagy occurrence. Moreover, ethanol-induced reduction in the number of autophagosomes and autolysosomes was recovered by KD, and CQ could further enhance the accumulation of autophagosomes in mCherry-EGFP-LC3 adenovirus-transfected AML12 cells, suggesting the promotive effects of KD on autophagy [108].

Except being a cargo receptor of autophagic degradation substrates, P62 is simultaneously recognized as an adaptor of Keap1 which can degrade Nrf2. Increasing P62 exhibits more potent competitiveness than Nrf2 for binding with Keap1 which leads to the release of Nrf2 from Keap1 and triggers Nrf2 activation, following anti-oxidation mechanisms. The regulation of the P62-Nrf2-Keap1 anti-oxidative pathway was described to be involved in FLD. A study has shown that Physalin B elicited preventive effects on NASH, which is at least partially associated with the activation of autophagy and the P62-Nrf2-Keap1 mediated anti-oxidative stress pathway [109]. The therapeutic effects of glycycoumarin and dihydromyricetin on ALD were also identified to be implicated in both autophagy induction and P62-Nrf2-Keap1-induced anti-oxidant activity in other studies [110,111]. Although these three compounds mentioned above could trigger autophagy as characterized by multiple methods, the role of P62 as an autophagy adaptor rather than its anti-oxidative effects and whether natural-product-induced clearance of LDs is related to autophagy induction in ALD remains to be elucidated in depth. Changing a way of thinking, the expression of P62 can not only be used to evaluate autophagy but also indicate the capacity of the endogenous redox system, which can be set as a novel direction for future research.

### 3.3. Effects of Natural-Product-Mediated Autophagy on Liver Fibrosis (LF)

As the shared pathological process occurs in almost all chronic liver diseases, LF is well documented as a compensatory mechanism to repair wounds in response to injuries. The typical characteristic of LF is an excessive accumulation of extracellular matrix (ECM) such as collagens, α-smooth muscle actin (α-SMA), and fibronectin which are all mainly derived from activated hepatic stellate cells (HSCs). As comprehensively summarized in a previous review, autophagy plays bidirectionally regulatory roles in the development of LF. On the one hand, autophagy activation has shown a positive correlation with LF progression through various bridging signaling pathways, including PI3K/Akt/mTOR, PDGF/TGF-β/Smads and β-arrestin 1/GSK-3β/SNAIL. The activation of autophagy can augment LF by inducing ductular reaction, activating HSC and accelerating the digestion of LDs. On the other hand, autophagy occurrence can protect against LF by inhibiting HSCs activation and pro-inflammatory cytokines secretion, as well as promoting LD regeneration. Manipulation of autophagy-related signaling pathways, containing mTOR/ULK1, JNK/c-JUN, Nrf2/Keap1, effectively protects against LF progress [112]. Therefore, the regulation of autophagy is a potential therapeutic target for reversing LF (Figure 3B).

It was previously confirmed that curcumin strongly protected against LF by inducing autophagy to blunt hepatocyte EMT. Increases in the autophagy-related proteins abundance and co-localization of LC3 and α-SMA/vimentin (indicated fibrosis) as well as the appearance of typical autophagy-like structures in the fibrosis model were observed after treatment with curcumin. The disruption of autophagy by CQ or Beclin1 siRNA compromised the anti-EMT effects of curcumin, which shed light on the significance of autophagy induction in alleviating LF. In terms of mechanism, experiments based on PPARα^−/−^, liver-specific ATG7-deficient mice or cells were performed and results suggested that curcumin could firstly induce ROS reduction by activating peroxisome proliferators-activated receptor-α (PPAR-α), and subsequently activated AMPK/mTOR-mediated autophagy. The TTC3d-SMURF2-SMADs signaling which was inextricably related to the occurrence and progression of EMT was blocked by activated autophagy finally [113]. As demonstrated in another previous study, Oroxylin A, a natural monoflavonoid isolated from *Scutellariae radix* was capable of protecting against LF by intensifying autophagy. 3-MA treatment completely abolished the anti-fibrotic effect of Oroxylin A, providing evidence that the protective effect of Oroxylin A on LF is dependent on the activation of autophagy [114]. In parallel, a widely distributed triterpenoid betulinic acid (BA) could alleviate fibrotic injury in an autophagy-dependent manner by inhibiting both the MAPK/ERK and AKT/mTOR pathways in vivo and in vitro. More abundant autophagosomes and autolysosomes were present in BA-treated HSCs compared with the control ones, as observed through autophagy tandem probe assay and TEM in which red spots and autophagy-like structures were measured. In addition, BA-induced up-regulation of LC3-II was accompanied by the down-regulation of α-SMA, which was potently impeded by 3-MA or BafA1, suggesting the involvement of autophagy in BA-mediated LF remission [115]. It was also unambiguously illustrated that Platycodin D (PD) from *Platycodon grandiflorum* blunted HSCs activation and development of LF by modulating the JNK/c-JUN signal pathway, which was termed as the upstream of autophagy/apoptosis occurrence. Although, PD was recognized to protect from LF and induce autophagy, apoptosis by promoting the JNK signaling pathway; however, whether liver fibrotic injuries are promoted or weakened when autophagy is inhibited or triggered separately is still absent in this study [116]. Moreover, purple *pitanga* extract from *Eugenia uniflora* L. was able to induce mitophagy, cell cycle arrest and apoptosis in GRX cells (a well-established activated HSC line) which was helpful in preventing the development of LF. The colocalization fluorescent signal between Mito-Tracker Green and Lyso-Tracker Red was enhanced, and more autophagosomes and autolysosomes were formed in purple *pitanga* extract-challenged GRX cells, demonstrating the mitophagy induction. Furthermore, they found that although purple *pitanga* extract removed damaged mitochondrial structures by inducing mitophagy, but failed to produce healthy replacements, which ultimately triggered GRX cells death and fought against LF. Nevertheless, the influence of purple *pitanga* extract on the cytotoxicity of GRX cells was not directly tested, and evidence of impaired mitochondrial regeneration after purple *pitanga* extract-induced mitophagy was also poor in this study. Therefore, it is unreasonable to conclude the effectiveness of purple *pitanga* extract on treating LF by regulating mitophagy [117]. Moreover, dihydrotanshinone I (DHI), a compound in *Salvia miltiorrhiza* Bunge, simultaneously decreased hepatic fibrosis indices and induced autophagy in BDL rats and HSCs by down-regulating the expression of YAP and inhibiting its interaction with TEAD2. The conversion of LC3-I to LC3-II was enhanced by DHI, which was further increased by CQ, representing the autophagy occurrence. However, there was a lack of any other results to indicate whether inhibiting autophagy would impair the beneficial effects of DHI on LF [118].

From the opposite side, autophagy plays a pro-fibrosis role in the liver. Qiaoting Hu et al. found that spleen tyrosine kinase (SYK) promoted HSCs activation by facilitating ROS-mediated autophagy occurrence, Silybin, a natural inhibitor of SYK, was termed as an antioxidant to abrogate the activation of HSCs and improve the excessive ECM accumulation in LF by intercepting ROS generation and blocking autophagy. Scavenging ROS by NAC abolished the down-regulation of autophagy in Silybin-treated cells, and supplemented with Silybin and SYK inhibitor GS-9973 could act synergistically against LF progression with good tolerance, which confirmed anti-fibrosis properties of Silybin through inhibiting autophagy [119]. Another previous study has demonstrated that SSd repressed oxidative stress-induced HSCs activation by up-regulating interactions between estrogen and its membrane receptor (G protein-coupled estrogen receptor, GPER1) and nuclear receptors (ER-α, ER-β). They observed that SSd could effectively alleviate fibrosis and down-regulate autophagy by recovering the expression of GPER1 in TGFβ1-treated LX2 cells and carbon tetrachloride (CCl_4_)-induced hepatic fibrosis mice. In the meanwhile, the role of the GPER1/autophagy pathway in SSd-induced LF alleviation was further validated, since autophagy activator RAPA and GPER1 antagonist G15 significantly impeded the promotive effects of SSd on liver fibrosis regression [120].

### 3.4. Potential Therapeutic Effects of Natural-Product-Mediated Autophagy on Viral Hepatitis

Viral hepatitis induced by various hepatitis viruse infections have taken place during the second half of the 20th century, and still exert a detrimental effect on the infected cells by both direct cytopathicity and immune-mediated mechanisms, causing acute and persistent infections associated with chronic inflammation that eventually progressing to liver cirrhosis and HCC [121,122,123,124]. On account of the huge population base of HBV/HCV infection, it is still extremely rigorous to fight against them. Roughly 30% of the world’s population show HBV infection in the present or past in line with the serological data, and an estimated 170 million persons represents an HCV-infected pandemic [125,126]. Although immunomodulatory drugs or antiviral agents usually aim to suppress virus replication currently, they were almost invariably unsuccessful owing to the very high rates of relapse and the less sustained response, and no effective cure is available yet [127]. Acting as a ubiquitous mechanism to maintain cell homeostasis, the discovery of autophagy provides insights into the treatment of viral hepatitis. Based on previous reports, we found that autophagy possessed multifaced roles in virus infection in liver [18]. Autophagy activation not only protects cells from virus infection-induced defects in lipid metabolism, but also antagonize innate antiviral immunity to enhance the virus replication [128,129]. Several natural products have been demonstrated to possess potential antiviral activities in liver by modulating autophagy.

In a previous study, they found that HBV infection could induce incomplete autophagy characterized by the promoted lipidation of LC3 and the disturbed degradation of P62 and GFP-LC3. HBV-induced incomplete autophagy significantly favored the intracellular replication of virus as supported by increased HBV titer in ATG5 and ATG7-depleted HepG2 and HepG2.2.15 cells. Efficacious as starvation-induced autophagy, Epigallocatechin-3-gallate (EGCG) opposed HBV-induced incomplete autophagy by recovering lysosomal acidification and was unfavorable for HBV replication. HBV transfection-induced impairment in the degradation of P62 and GFP-LC3 were also simultaneously recovered in the presence of EGCG [130]. Clinical data have shown that HBV-specific T cell function, mitochondrial and proteasomal functions as well as autophagy were impaired in patients with chronic HBV infection. The recoveries in the function of T cells to respond to HBV virus and protein homeostasis when treated with polyphenols such as resveratrol and oleuropein were observed. Nevertheless, whether autophagy recovery is responsible for the application of polyphenols in the treatment of HBV infection is still not explicitly elucidated which may result from the insufficiency of autophagy activity [131]. Another previous study revealed that a natural compound isolated from *Mundulea sericea* (*Leguminosae*), deguelin, could inhibit autophagy initiation, a cellular machinery required for HCV replication, and then damaged HCV replication and decreased cell viability in HCV-infected Huh7 cells. Autophagy activation by over-expressing Beclin1 diminished the pharmacological effects of deguelin, whereas autophagy deficiency mediated by Beclin1 knockout exhibited the opposite effects, suggesting the significance of autophagy in properties of deguelin to HCV infection [132].

### 3.5. Potential Therapeutic Benefits of Natural Products on Acute Liver Injury (ALI) by Modulating Autophagy

**Figure 4:**
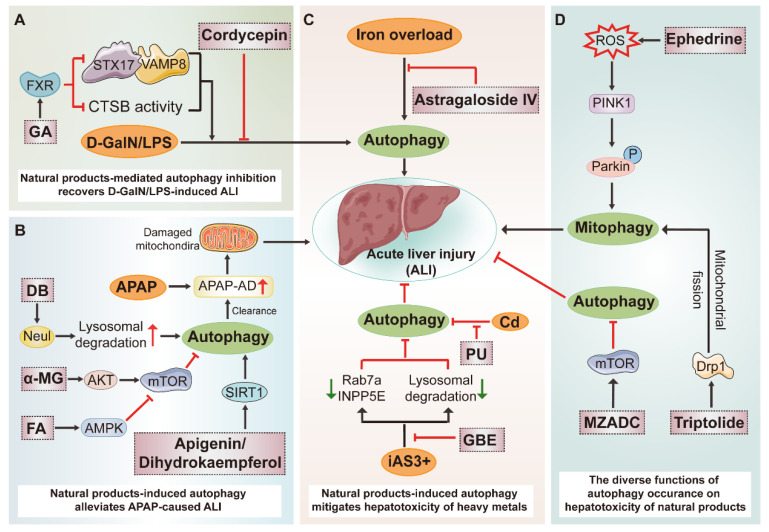
Potential therapeutic benefits of natural products on ALI by modulating autophagy. (**A**) The protective functions of natural-product-induced autophagy in D/L-induced ALI. GA, 18β-Glycyrrhetinic-acid. (**B**) Natural-product-mediated autophagy activation promoted the clearance toxic metabolite (APAP-AD) of APAP. DB, dipsacoside B; α-MG, alpha-mangostin; FA, ferulic acid. (**C**) The alleviation of natural products to hepatotoxicity of heavy metals by triggering autophagy. PU, puerarin; GBE, *ginkgo biloba* extract. (**D**) The different effects of autophagy on the Chinese-herb-induced liver injury. MZADC, the methanol extract of *Zanthoxylum armatum* DC.

As a pleiotropic metabolic organ, the liver is predisposed to suffer from damages when exposed to an overdose of drugs, unhealthy life style and detrimental living circumstances. Multiple factors can result in acute liver injury (ALI), and eventually lead to liver failure, which is rare but life-threatening. Natural products possess varied regulatory abilities to ALI by intervening autophagy (Figure 4).

An overdose of acetaminophen (APAP) is considered to be the most common medical factor used to induce ALI and related mortality. N-acetyl-P-phenylpropanimide (NAPQI), the bioactive metabolic intermediate of APAP, is a major toxic substance which can be generally degraded by hepatic GSH. However, over-accumulation of NAPQI will deplete GSH and bind with intracellular molecules such as mitochondrial protein, then forming APAP protein adduct (APAP-AD), subsequently resulting in a spectrum of adverse cascade reactions. Wen-Xing Ding et al. revealed that removal of APAP-AD by autophagy protected against APAP-induced liver injury in mice. When administrated with an overdose of APAP, the autophagy adaptor p62 was recruited to APAP-AD, targeted to autophagosomes and was then degraded in autolysosomes. At the same time, autophagy blockage was coupled with both the accumulation of APAP-AD and the aggravation of liver injury, highlighting the protective role of autophagy in APAP-induced liver damage [133]. Natural product dipsacoside B (DB) was identified to protect against APAP-induced liver injury through augmenting autophagy-mediated removal of excessive APAP-AD which was measured by Poncea red staining in vivo and in vitro. Mechanistically, the decline of Neu1, which is mainly located in the lysosome and acts as a negative regulator of lysosome exocytosis, is closely connected to the clearance of DB-triggered APAP-AD deposition [134]. Additionally, dihydrokaempferol and apigenin were determined to ameliorate APAP-induced liver injury partially by anchoring to and activating SIRT1, a positive regulator of autophagy, in APAP-treated C57BL/6 mice and L-02 cells. Decrease in the expression of SIRT1 was recovered and autophagy activation was further enhanced by dihydrokaempferol and apigenin in APAP-treated mice or cells. In the meanwhile, SIRT1 inhibitor EX-527 exacerbated APAP-induced hepatotoxicity and inhibited autophagy generation [135,136]. Furthermore, ferulic acid (FA) isolated from multiple herbs was also reported to ameliorate APAP-induced ALI by promoting the AMPK-mediated protective autophagy. The addition of AMPK inhibitor compound c abolished FA-induced up-regulation of autophagy activation-associated proteins such as LC3, ATG5 and eventually the hepatoprotective activity of FA [137]. From a different perspective, a researcher found that APAP could induce harmful overactivation of autophagy, which could be rectified by alpha-mangostin (α-MG) derived from *Garcinia mangostana* by activating the AKT-mTOR pathway. Unfortunately, there remains a paucity of direct proof to manifest how APAP-induced liver injury develops when autophagy is intercepted or facilitated [138]. Except for chemical drugs, it is worth noting that many herbs and dietary supplements have also been regarded as a major cause of drug-induced liver injury (DILI) worldwide which extremely limits their therapeutic implications. As a pivotal toxic mechanism of herb-induced liver injury (HILI), autophagy is served as a potentially therapeutic strategy for HILI [139]. The hepatotoxicity of triptolide has long been concerned and previous research indicated that mitophagy induction triggered by Drp1-mediated mitochondrial fission were closely related to its hepatotoxicity. In triptolide-treated L02 cells, the abundance of mitophagy-related proteins LC3-II, Drp1, P62, Mfn1 and fluorescence colocalization between Mito-Tracker green and Lyso-Tracker red were measured. Moreover, a selective Drp1 inhibitor, Mdivi-1, possessed an inhibitory effect on triptolide-induced mitophagy and comprised its hepatotoxicity [140]. The liver toxicity of ephedrine, derived from Chinese ephedra, should not be ignored, since it was widely used in clinical practice. A recent study has shown that ephedrine activated mitophagy and destroyed mitochondrial function in a ROS-relied manner, which accounted for its suppressive impacts on LX-2 cells viability. The mitophagy induction by evoking the PINK1-Parkin pathway was determined after ephedrine treatment, and was abrogated by knockout of Parkin or clearage of mitochondrial ROS using Mito TEMPO. On the other hand, whether ephedrine-induced mitophagy is beneficial to the recycling of damaged mitochondria or otherwise induces ACD is still unknown [141]. As major bioactive ingredients of *Radix bupleuri* root (chai hu), Saikosaponin (SS) a and SSd were also major toxic ingredients in it. Shifeng Wang et al. reported that SSa/SSd bound and inhibited the activity of sarcoplasmic/endoplasmic reticulum calcium ATPase (SERCA), and subsequently induced autophagy in HepG2 cells. Blocking autophagy by 3-MA significantly potentiated SSa/SSd-induced cell toxicity, implying that SS-induced autophagy activation may act as a protective feedback mechanism [142]. Moreover, the latest study demonstrated that the methanol extract of *Zanthoxylum armatum* DC. (MZADC) induced liver damage through mTOR/ULK1-meidated autophagy suppression which subsequently induced intracellular oxidative damage. They observed that MZADC inhibited the lipidation of LC3-I and degradation of P62. MZADC-induced autophagy reduction and oxidative damage were significantly recovered in BRL 3A cells when pretreated with RAPA [143].

In experimental studies, D-galactosamine/Lipopolysaccharide (called D/L latter) is commonly used to establish ALI or ALF mouse models. Recently, GA, the main active metabolite of licorice, was reported to protect from D/L-induced ALI in vivo and TNF-α + SM-164 (TS)-induced ALI in vitro by recovering the expression of Pregnane X receptor (PXR) which inhibited the autophagosome–lysosome fusion and lysosomal function while having no influence on autophagy initiation. Compared with the D/L group, the interactions between fusion-related protein Stx17 and Vamp8, as well as their expression, were remarkably decreased, and mRNA levels of lysosomal genes such as CstB, and TPP1 were also reduced, followed with the over-accumulation of autophagy substrate proteins including LC3-II and P62 in D/L plus the GA group, which was in accordance with the D/L plus CQ group. Moreover, in D/L-treated C57BL/6 mice, the depletion of PXR caused more serious hepatocyte apoptosis and liver damages, which abolished impacts of GA on autophagy, and the alleviation of ALI. However, they also found that 3-MA weakens the protective effects of GA on D/L-induced ALI. For this reason, the autophagosome–lysosome fusion which is termed as a critical part of an intact autophagy flux may not be the main target of GA or is described indistinctly in this study [144]. Jin Li et al. reported that Cordycepin, the major active ingredient of *Cordyceps militaris*, was documented to alleviate inflammation, oxidative stress and autophagy overactivation in D/L-induced ALF. Autophagy inhibition induced by Cordycepin was observed; however, the significance and necessity of autophagy for its protective effects against ALF were not clearly clarified [145]. Moreover, puerarin administration which derived from *radix puerariae* was also reported to facilitate autophagy and suppress cells apoptosis in D/L-treated mice, which only concluded from the levels of related proteins; however, mechanisms by which autophagy recovery regulated liver damage was not entirely clear [146]. In another study, the therapeutic and hepatoprotective effects of Withaferin A (WA), isolated from the herb *Withania Somnifera*, on D/L-induced ALI were well evaluated. Although WA could restore the autophagy defects in D/L-treated mice, 3-MA failed to abolish the hepatoprotective influence of WA, which represented autophagy and was dispensable for the advantageous effect of WA [147].

For heavy-metal-induced liver toxicity, diverse autophagy regulators were tested for their therapeutic potential. Arsenite (iAs3+) exhibits hepatic toxicity by thoroughly interfering with multiple steps of autophagy. Specifically, iAs3^+^ not only stimulates autophagosome synthesis via inhibiting the E2F1/mTOR pathway, which is similar with RAPA, but also simultaneously blocks autophagosome degradation via suppressing the expression of fusion-related proteins INPP5E and Rab7 and destroying the lysosomal activity. Additional challenges with autophagy inducer RAPA, inhibitor CQ or a combination of the two facilitated iAs3^+^-induced cytotoxicity, while disrupting the formation of autophagosomes by 3-MA showed protective effects, which further revealed the significance of autophagy in iAs3^+^-induced hepatotoxicity. In the meantime, results also indicated that *ginkgo biloba* extract (GBE) could significantly alleviate the toxicity of iAs3+ in part by recovering damaged autophagosome–lysosome fusion and lysosomal degradation [148]. Cadmium (Cd) is another common heavy metal contamination which is highly toxic to the liver, and puerarin (PU) isolated from the root of the *Pueraria lobata* could effectively alleviate Cd-induced apoptosis and restore the impaired autophagic flux in AML-12 cells. In detail, Cd-induced autophagy inhibition activity was analogous to CQ as the Cd-treated group and the Cd plus CQ group exhibited similar accumulation of autophagosomes, and the above impacts were greatly reversed by PU. However, exactly how is autophagy involved in the toxicity of Cd remained fuzzy [149,150]. There is consensus that iron overload is always companied by dysregulation of multiple cellular metabolic pathways such as redox homeostasis, iron handling and various energy metabolism as well as autophagy activation, which is closely implicated in various human diseases [151]. An early study demonstrated that ferritinophagy, a selective autophagy is recognized to specifically degrade the cellular iron storage proteins and frritinophagy cargo receptor NCOA4. Additionally, multiple autophagy-related genes are positively correlated with ferroptosis occurrence, an iron-dependent form of regulated necrosis. Blockage of ferritinophagy by inhibiting autophagy or depleting NCOA4 abolishes the accumulation of ferroptosis-associated cellular iron and ROS, in addition to eventual ferroptotic cell death [152]. As a result, autophagy can be regarded as an attractive target for iron-overload-induced hepatic injury. The therapeutic potential of astragaloside IV to iron-overload-induced L02 cells apoptosis by suppressing the excessive activation of autophagy was reported early. It is necessary to account for the regulatory mechanism of astragaloside IV-modulated autophagy on iron overload within the next in vivo study [153].

## 4. Discussion

**Figure 5:**
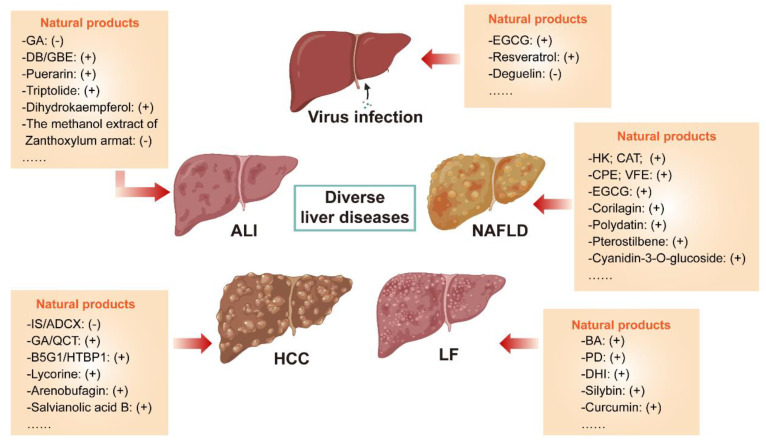
Natural products ameliorate liver diseases by modulating autophagy. (+) indicates autophagy activation; (-) represents autophagy inhibition.

Merging findings have highlighted that multiple natural products exert regulatory effects on autophagy during the treatment of liver diseases. It is easy to recognize that the role of natural-product-mediated autophagy in liver diseases is complex (Figure 5). Some natural products, similar to RAPA, promote or inhibit the nucleation of phagophores and formation of autophagosomes by regulating the PI3K/AMPK/mTOR pathway in liver diseases, and the above autophagy induction can be reversed by 3-MA [55,90,113]. Similar to the effects of BafA1 or CQ, some natural products inhibit autophagosome–lysosome fusion by impairing the acidic environment of lysosome and decreasing the expression of fusion-related protein Rab7a, subsequently alleviating liver dysfunction [57,144,148]. Some others are able to recover or destroy the lysosomal degradation function by intervening the transcription of TFEB or lysosomal acid hydrolase activity, and thus modulating liver homeostasis [59,97,130]. Moreover, some natural products have impacts on more than one part of autophagy flux. For instance, PB stimulated ER stress by increasing autophagosomes but blocking degradation to modulate proteostasis in HCC [56]. PGG induced HCC senescence, which relied on the autophagosome formation, while inhibiting autophagic degradation which results in HCC apoptosis [70]. For the paradoxical role of autophagy in diseases, especially cancers, basal autophagy generally suppresses tumor initiation. However, in most cases examined so far, autophagy facilitates tumor progression because of differences in the timing and context of tumor development [154]. In addition, emerging from different models, some natural products exhibited opposite effects on regulating autophagy. For instance, SSd-mediated autophagy inhibition was reported to be responsible for its hepatotoxicity [142], whereas in another study, SSd increased the radiosensitivity of HCC cells by inducing autophagy [64]. Moreover, BBR dampened the autophagic response in HCV-infected Huh-7 cells, and pharmacological inhibition of autophagy conversely augmented BBR-induced cell death [75]. On the other hand, BBR was also suggested to induce ACD, which could be significantly prevented by 3-MA [76].

**Figure 6:**
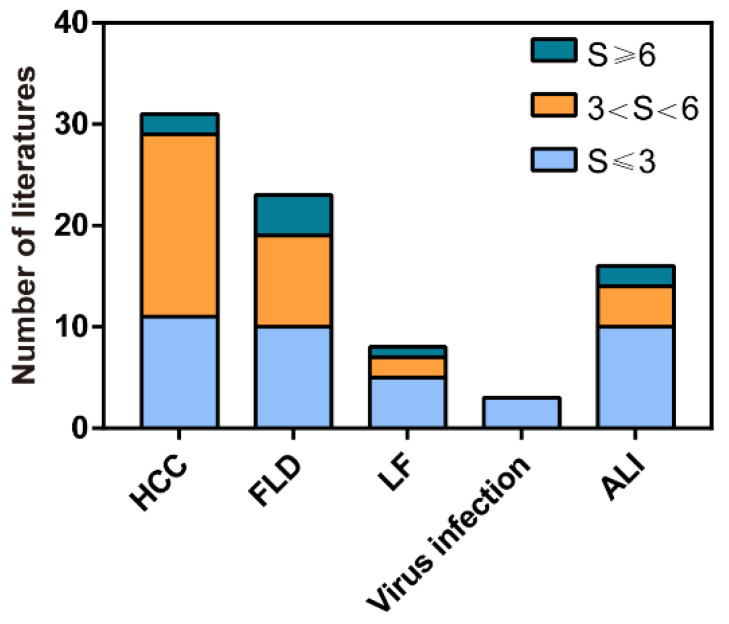
The score of reviewed studies. The abscissa shows the type of liver diseases and the ordinate indicates the total number of studies involved in different score segments. S, score.

The multistep processes of autophagy flux and complex roles of autophagy in diverse diseases (either protective or pathogenic) limit pharmacological studies of autophagy regulators to a great extent. Herein, we put forward a scoring system based on the strength of evidence to analyze the completeness and scientific significance of the current literature for reference. The scoring details are as follows: (1) If the expressions of protein, mRNA and fluorescence of autophagy markers were evaluated, 0.5 points were obtained each. (2) If the degradation of autophagy substrates was involved, 0.5 points were obtained. (3) If the autophagic ultra-structures and colocalization between different markers associated with varied autophagy-related structures were implicated, one point was obtained each. (4) If the underlying mechanisms or some novel specific mechanisms connecting autophagy regulation were elucidated deeply, one point was obtained each. In terms of the above system, the in vivo and in vitro scores were collected, leading to a final score for every paper reviewed above, as shown in Figure 6.

According to this score system, we found that, in almost half of these studies, the authors failed to provide convincing and consistent evidence to elucidate the regulatory effects and underlying mechanisms, which brought significant obstacles to develop autophagy-regulating natural products as novel treatments for liver diseases. Applying novel methods and rigorous experimental design are indispensable in the future research [155]. The crucial role of natural-product-mediated autophagy in the treatment of liver diseases, the underlying molecular mechanisms, as well as representative scores of reviewed literatures based on the above scoring system are illustrated in Table 1.

## 5. Conclusions

Over the last several decades, tremendous advances have been made in the discovery of therapeutic approaches for liver-related disorders, and in which regulating autophagy is proposed as a novel strategy. Although controversial conclusions were drawn by using different experimental objects and employing varied technical approaches in previous studies, we still believe that by addressing the above-mentioned issues, autophagy-regulative natural products are valuable drug candidates with promising prospects for the treatment of liver diseases.

## Figures and Tables

**Table 1:** The regulatory effects of natural products and scores for studies.

Disease	Natural Product	Resource	Autophagy			Molecular Mechanism	Score
			Autophagy Flux	Autophagosome Information	Degradation in Autolysosome		
HCC	ADCX	*Cimicifugae rhizoma*	(-)		(-)	NA	7.5
	IS	*Epimedium koreanum* Nakai	(-)		(-)	NA	5
	RA-XII	*Rubia yunnanensis*	(-)	(-)		Inhibiting the AMPK-mTOR pathway	3.5
	PB	*Polygonum bistorta* L.	(-)		(-)	NA	2.5
	6-shogaol	Ginger	(-)		(-)	NA	2
	GN	*Schisandra chinensis* (Turca.) Baill	(-)	(-)		Activating the PI3K-AKT pathway	1.5
	QCT	Wide distribution	(+)	(+)		Inhibiting the AKT-mTOR pathway	5
	Uvangoletin	*Sarcandra glabra* (Thunb.) Nakai	(+)	(+)		Inhibiting the AKT-mTOR pathway	4.5
	rBTI	Tartary buckwheat	(+)	(+)		NA	4.5
	XAG	*Angelica keiskei* (Miq.) Koidz.	(+)	(+)		Activating the AMPK-mTOR pathway	4
	PGG	*Rhus chinensis Mill*	(+)	(+)		NA	3.5
	SSd	*Radix bupleuri* root	(+)	(+)		Inhibiting the mTOR pathway	3
	DHA	*Artemisia annua* L.	(+)	(+)		Promoting the ROS production	3
	SK	*Lithospermum erythrorhizon*	(+)	(+)		Down-regulating PYCR1, Inhibiting the AKT-mTOR pathway	3
	Bufalin	Bufonid’s skin	(-) or (+)	(-) or (+)		NA	2–4
	BBR	*Berberis aristata*	(-) or (+)	(-) or (+)		NA	1–3
	SM	*Solanum nigrum* L.	(+)	(+)		NA	1.5
	GA	*Glycyrrhiza uralensis Fisch*	(+) *	(+)		NA	5.5
	Lycorine	*Lycoris radiata*	(+) *	(+)		NA	5.5
	Arenobufagin	Bufonid’s skin	(+) *	(+)		NA	5
	HTBPI	Tylophora ovata	(+) *	(+)		NA	4.5
	7-Deoxynarciclasine	*Lycoris radiata*	(+) *	(+)		NA	4.5
	Salvianolic acid B	*Salvia miltiorrhiza* Bunge	(+) *	(+)		Inhibiting the AKT-mTOR pathway	4.5
FLD	Pterostilbene	Wide distribution	(+)	(+)		NA	7.5
	VFE	*Valeriana fauriei* Briq	(+)	(+)		Inhibiting the mTOR-ULK1 pathway	6.5
	Corilagin	Wide distribution	(+)	(+)		NA	6
	Cyanidin-3-O-glucoside	Black soybean testa	(+)	(+)		Activating the PINK1-Parkin pathway	6
	Polydatin	*Polygonum cuspidatum* Sieb. et Zucc.	(+)	(+)	(+)	Activating the TFEB and inhibiting the mTOR	5
	EGCG	Green tea	(+)	(+)		Activating the AMPK	5
	CAT	*Rehmannia glutinosa* DC.	(+)	(+)	(+)	Activating the AMPK-TFEB pathway	5
	HK	*Magnolia* genus L.	(+)	(+)		Activating the SIRT3-AMPK pathway	4.5
	CPE	*Cyclocarya paliurus*	(+)	(+)		NA	4.5
	RPS	*Capsicum annuum* L.	(+)	(+)		Activating the AMPK	4
	Ginsenoside Rb2	*Panax ginseng* C.A.Mey.	(+)	(+)		Activating the SIRT1-AMPK pathway	4
	KD	*Anoectochilus roxburghii* (Wall.) Lindl. (Orchidaceae)	(+)	(+)		Activating the STRAD/LKB1-AMPK pathway	3.5
	IA	*Salvia miltiorrhiza* Bunge	(+)	(+)	(+)	Activating the TFEB	2
	BPF	*Citrus bergamia* Risso et Poiteau	(+)	(+)		NA	1.5
LF	Curcumin	*Curcuma zedoaria* Roxb and…	(+)	(+)		Activating the AMPK-mTOR pathway	7
	BA	Wide distribution	(+)	(+)		Inhibiting the MAPK/ERK pathway	5.5
	Silybin	*Silybum marianum* (L.) Gaertn.	(-)	(-)		Inhibiting the ROS production	3.5
	Oroxylin A	*Scutellariae radix*	(+)	(+)		Inhibiting the MAPK-ERK and AKT-mTOR pathways	3
	PD	*Platycodon grandiflorum.*	(+)	(+)		Inhibiting the JNK/c-JUN pathway	3
	Purple pitanga extract	*Eugenia uniflora* L.	(+)	(+)		NA	3
	SSd	*Radix bupleuri* root	(-)	(-)		Recovering the expression of GPER1	3
	DHI	*Salvia miltiorrhiza* Bunge	(+)	(+)		Down-regulating the expression of YAP	2.5
Virus infection	EGCG	Green tea	(+)	(+)		Recovering lysosomal acidification	3
	Deguelin	*Mundulea sericea* (Leguminosae)	(+)	(+)		NA	2
	Resveratrol	*Polygonum cuspidatum* Siebold and Zucc.	(+)	(+)		NA	1
	Oleuropein	*Canarium album* (Lour.) DC.	(+)	(+)		NA	2
ALI (APAP-induced-ALI)	Dihydrokaempferol	Wide distribution	(+)	(+)		Activating the SIRT1-AMPK pathway	3.5
	Apigenin	*Matricaria chamomilla*	(+)	(+)		Activating the SIRT1-AMPK pathway	3.5
	FA	Wide distribution	(+)	(+)		Activating the AMPK	3
	DB	*Lonicera acuminata* Wall.	(+)	(+)		Down-regulating the Neu1	2.5
	α-MG	*Garcinia mangostana*	(-)	(-)		augmenting the AKT-mTOR pathway	1
ALI (Chinese-herb-induced ALI)	Triptolide	*Tripterygium wilfordii Hook. f.*	(+) ^#^	(+)		Promoting the Drp1-mediated mitochondrial fission	5.5
	Ephedrine	Chinese ephedra	(+) ^#^	(+)		Evoking the PINK1-Parkin pathway	4
	MZADC	*Zanthoxylum armatum* DC.	(-) ^#^	(-)		Activating the mTOR-ULK1 pathway	2
	SSa/SSd	*Radix bupleuri* root	(+) ^#^	(+)		Inhibiting the activity of SERCA	2
ALI (D/L-induced ALI)	GA	*Glycyrrhiza uralensis Fisch*	(-)		(-)	Recovering the expression of PXR	6
	Puerarin	*Radix puerariae*	(+)	(+)		NA	2.5
	Cordycepin	*Cordyceps militaris*	(-)	(-)		NA	1.5
	WA	*Withania Somnifera*	(+)	(+)		NA	1
ALI (heavy-metal-induced ALI)	GBE	*Ginkgo biloba*	(+)	(+)		Inhibiting the E2F1/mTOR pathway	8
	Astragaloside IV	*Astragalus aaronsohnianus* Eig	(-)	(-)		NA	1.5
	PU	*Pueraria lobata*	(+)	(+)		NA	1

Note: (-) and (+) represent autophagy inhibition and autophagy activation, respectively, which also indicate that natural-product-mediated autophagy inhibition and activation are positively related to its therapeutical effects on liver diseases. (+) * shows that natural-product-induced autophagy activation is regarded as a side effect associated with their anti-HCC activities. (+) ^#^ and (-) ^#^ indicate that natural-product-modulated autophagy induces hepatotoxicity. NA, Not Applicable.

## Data Availability

Not applicable.

## Abbreviations

DFCP1	double FYVE domain–containing protein 1
ULK	Unc51-like kinase
PI3K	phosphatidylinositol 3-kinase
LC3	microtubule-associated protein light chain 3
FIP200	FAK family kinase-interacting protein of 200 kD
VAPA/VAPB	(Vesicle-associated membrane protein)-associated Protein A/B
VPS34	vacuolar protein sorting 34
IMATs	isolation membrane-associated tubular structures
ERGIC	ER-Golgi intermediate compartment
ESCRT	endosomal sorting complexes required for transport
SNARE	endosomal sorting complexes required for transport
HOPS	homotypic fusion and protein sorting
AMPK	Adenosine 5‘-monophosphate (AMP)-activated protein kinase
AKT	Protein kinase B
mTOR	mammalian target of rapamycin
MAPK	mitogen-activated protein kinase
PINK1	PTEN-induced kinase 1
Keap1	Kelch-like ECH-associated protein 1
Nrf2	erythroid 2-related factor 2
ERK	extracellular regulated protein kinases
LPS	lipopolysaccharide
D-GalN	D-galactosamine
